# The Barriers and Enablers to Participation in Oncology Clinical Trials for Ethnically Diverse Communities: A Qualitative Systematic Review Using Metaethnography

**DOI:** 10.1097/NCC.0000000000001399

**Published:** 2024-11-21

**Authors:** Lorraine Turner, Sally Taylor, Ashleigh Ward, Fiona Thistlethwaite, Janelle Yorke

**Affiliations:** Author Affiliations: Department of Research & Innovation (Ms Turner) and Christie Patient Centred Research (Dr Taylor, Ms Ward and Professor Yorke), The Christie NHS Foundation Trust; School of Nursing, Midwifery and Social Work, The University of Manchester (Ms Turner and Professor Yorke); Department of Medical Oncology, The Christie NHS Foundation Trust (Professor Thistlethwaite); and Division of Cancer Sciences, School of Medical Sciences, The University of Manchester (Professor Thistlethwaite), Manchester, United Kingdom.

**Keywords:** Cancer, Clinical trial, Ethnicity, Inequalities, Participation, Qualitative, Race, Research, Underrepresentation

## Abstract

**Background:**

Racial and ethnic inequities exist in cancer clinical trial participation. Low recruitment across ethnically diverse communities contributes to health inequalities further disproportionately affecting these groups. Understanding barriers and enablers to clinical trial participation for ethnic minorities is key to developing strategies to address this problem.

**Objective:**

To explore, evaluate, and synthesize qualitative research surrounding patients’ lived experiences and perceptions of participating in cancer clinical trials from ethnically diverse groups.

**Methods:**

Noblit and Hare’s 7-stage metaethnography was used. Seven databases were searched. Inclusion criteria were as follows: qualitative studies published in English from January 1, 2012, to January 31, 2022; patients from any ethnic minority 18 years and older with a cancer diagnosis; and cancer patients’ carers and healthcare professionals (HCPs)/healthcare leaders involved in the delivery of cancer clinical trials.

**Results:**

The majority of included articles were conducted in the United States. Interpretive qualitative synthesis resulted in 7 categories including patient perceptions and beliefs and HCP perception of trial burden and social determinants of health. Four lines of argument were established.

**Conclusions:**

The findings capture the experience and perceptions of ethnic minority patients, their carers, HCPs, and healthcare leaders in this area of research. Incongruities exist between patient-reported barriers and those perceived by HCPs. Published empirical research outside the United States is limited.

**Implications for Practice:**

When developing strategies to increase clinical trial participation, research literacy, cultural safety, and unconscious biases within healthcare need to be addressed. Further research to examine intersectionality and the role of faith in decision-making among ethnic groups is warranted.

Oncology clinical trials designed to test novel cancer therapies, progress the science of cancer, and develop new and advanced treatments are essential to improve outcomes for patients with cancer.^1^ With greater participation, clinical trial outcomes will be more timely, efficient, and accessible. Cancer trials offer patients an additional treatment option and the opportunity to access treatments not routinely available.^2^ Equity of access and increased diversity in randomized clinical trials increase opportunities for discovering effects that may be particularly relevant to diverse communities and ensure results are generalizable to the population affected.^3^^4^

Only a small percentage (approximately <5%–8%) of eligible adult cancer patients participate in trials.^5^ Recruitment and retention are lower for cancer patients from ethnic minorities.^6^^7^^8^ Many factors, broadly classified as physician, patient, and system-related,^5^^6^^9^ can influence clinical trial participation. Within these broad categories lie complex and multifaceted influences that affect patients, physicians, and healthcare providers’ decision-making around this phenomenon. A greater understanding of the barriers to and facilitators of clinical trial participation across underrepresented populations is paramount to developing strategies to improve trial recruitment and retention.^8^

Using qualitative methodology allows analytical depth and contextual detail to help understand participants’ views, attitudes, beliefs, and experiences and contribute to a greater understanding of the phenomena that are being questioned.^10^ Previous systematic reviews have focused on defining and quantifying clinical trial barriers such as trial availability across various health systems, eligibility criteria, and reported opportunities for decision-making.^5^^11^ Others have included randomized controlled trials across all types of trials including prevention and treatment.^6^^12^

## Aim

The aim was to explore, evaluate, and synthesize the qualitative research surrounding patients’ lived experiences and perceptions of clinical trial participation from ethnically diverse groups and to understand the views and perceptions of carers, healthcare professionals (HCPs) and healthcare leaders (HCLs) of the barriers to and facilitators of ethnic minorities participating in oncology trials.

## Methodology

This study was a metaethnography review of the literature. eMERGe^13^ guidance was followed throughout. Its protocol is registered on PROSPERO (ID: CRD42022330829). The 7 stages of metaethnography developed by Noblit and Hare were followed.^14^

### Search Strategy and Screening

An initial comprehensive scoping search helped to refine the research question and develop the search terms for the main review. Table 1 shows the search terms used. Key words were used across 7 databases, adapting the Boolean operators and MeSH vocabulary in collaboration with an information specialist librarian. The bibliographic databases included Ovid MEDLINE, Ovid EMBASE, CINAHL Plus, PsychINFO, ASSIS (Applied Social Sciences Index and Abstracts), Social Sciences Index, and The Cochrane Library. Inclusion criteria are shown in Table 2. Eligibility criteria were broad to gain insight from patients and their family members/carers who may play a key role in decision-making. It was deemed important to capture reciprocal and refutational concepts between patients and HCPs. Titles and abstracts were screened independently by 2 reviewers (L.T., A.W.). The screening and selection tool was piloted on 20 abstracts; any adjustments to the screening and selection tool were made at this point. Articles that met the inclusion criteria were read in full, and the reason for exclusion was recorded. Any uncertainty or discrepancies were resolved through discussions with a third member of the team, S.T.

**Table 1:** Search Strategy Including Search Terms

Oncology	Barriers/Enablers	Participation	Clinical Trials	Study Type
(tumo?r* OR cancer* OR neoplas* OR oncolog* OR melanom* OR maligna* OR gliom* OR carcin* OR sarcom* OR metasta* OR chemotherap* OR radiotherap* OR leuk?em*) *NEOPLASMS/	((Socioeconomic OR cultur* OR rac* OR ethnic* OR religio* OR belie* OR depriv*) ADJ2 (factor* OR barrier* OR underserv* OR under-serv*)) (inequalit* OR disparit*) (Immigrat* OR migrant*) “Language barrier*” ((Barrier* OR Restrict* OR Restrain* OR Limit* OR Prevent* OR Declin*) ADJ2 (Participat* OR Enroll* OR Select* OR Recruit*)) Underrepresent* OR under-represent* OR “under represent*” exp SOCIOECONOMIC FACTORS/exp RACE FACTORS /exp HEALTHCARE DISPARITIES/exp HEALTH EQUITY/exp HEALTH DISPARITIES/	(Participat* OR enroll* OR select* OR recruit*) “Clinical Trial*” ADJ2 Recruit* OR Select* OR Participat* “Clinical trial* recruitment” Willing* ADJ2 participat* “Health literacy” OR “Health illiteracy” OR “Patient education” exp PATIENT SELECTION/ exp PATIENT EDUCATION AS TOPIC/	(“early phase” ADJ2 “clinical trial*”) (“Phase 1” OR “Phase 2” OR “Phase 3” OR “Phase I” OR “Phase II” OR “Phase III” OR “Phase One” OR “Phase Two” OR “Phase Three” ADJ2 “Clinical trial*”) (Clinic* OR Genomic* OR Biomedic* OR Pharmacogenomic* ADJ2 (Trial* OR Stud*)) exp CLINICAL TRIALS AS TOPIC/	“Qualitative” OR “Grounded theor*” OR “Focus group*” OR “Ethnography” NOT quantitative exp QUALITATIVE RESEARCH/ ANTHROPOLOGY, CULTURAL.mp exp FOCUS GROUPS/

Databases: Ovid MEDLINE, Ovid EMBASE, EBSCO CINAHL Plus, ProQuest PsychINFO, ASSIS (Applied Social Sciences Index and Abstracts), Social Sciences Index, and The Cochrane Library. One set of results per database. Format of results: RIS. Data limit: 01/01/2012+; investigate percentage published by year.

**Table 2:** Inclusion Criteria

• Studies published in English from January 1, 2012, to January 31, 2022
• Patients from any ethnic minority, 18 y and older, with a cancer diagnosis of any type and stage, actively (neoadjuvant, adjuvant, and palliative) or not actively undergoing therapeutic treatment
• Cancer patients’ family/carers, healthcare professionals, and healthcare leaders involved in the care of adult cancer patients and/or delivery of cancer clinical trials
• Studies using qualitative methodology/philosophies such as interviews, personal narratives, focus groups, participant observation, phenomenology, grounded theory, and ethnography
• Qualitative research undertaken in university/academic and local community hospitals, community settings, clinical trial outreach centers, and cancer centers within the United Kingdom and internationally

### Data Extraction and Synthesis

Prior to data extraction, quality appraisal using The Joanna Briggs Institute Critical Appraisal Checklist for Qualitative Research was independently completed by L.T. and A.W. “Raw data” from each article, including study and population characteristics, first-order constructs (research participants quotations), and summary of second-order constructs (metaphorical concepts interpreted by primary authors), were extracted and stored on an Excel spreadsheet (Microsoft Corp, Redmond, Washington).

L.T. and A.W. grouped first-order verbatim under concepts to represent an experience and repeated for each included article. These were then juxtaposed against each study to examine the relationships between the concepts to create common and recurring themes. The themes were then clustered into relevant categories. Starting with the most recently published article, the concepts were compared with each of the other articles in turn. Concepts were recorded as reciprocal (common/mutual findings) or refutational (divergence, opposing/contradictory findings). This process was iterative, with some categories being merged, or moved, and some new concepts created. The raw data were referred to when deciphering the relationships between concepts across the articles. Two separate syntheses were produced: one for patients/family or carers and one for HCPs including HCLs. Reciprocal and refutational themes across the 2 groups were identified, and thematic groups became distinct categories.

Categories and related concepts were displayed in color codes to help show reciprocal themes across the 2 population groups. Refutational syntheses were noted separately and included within the color concepts. Third-order constructs (a higher-order interpretation) from tertiary analysis of first- and second-order constructs were developed to produce a conceptual framework. Lines of argument from the third-order constructs were developed. Metaethnography has allowed the authors to synthesize and reinterpret conceptual data from primary studies to develop a higher-level, conceptual understanding around this complex phenomenon.

## Results

### Study Characteristics

**Figure 1:**
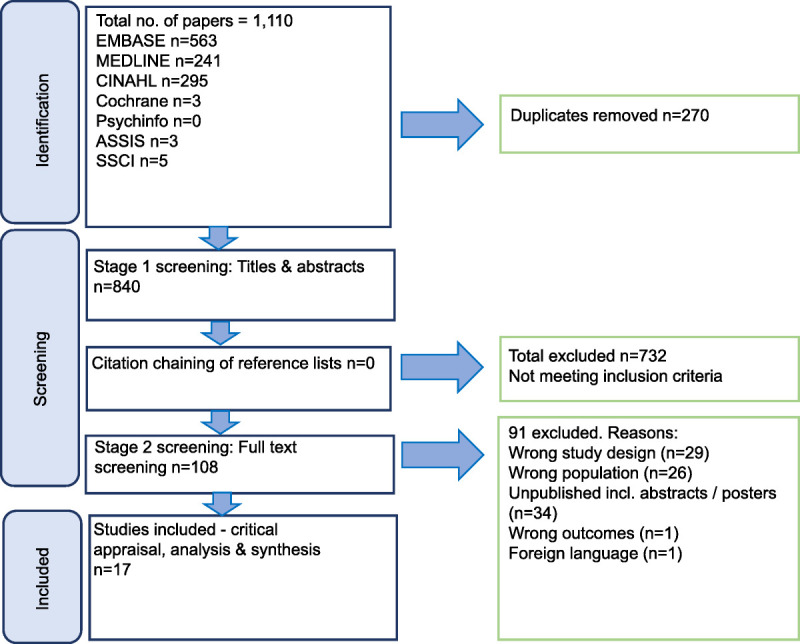
Results of searching, screening and selection using PRISMA guidelines. Figure designed using Microsoft Office PowerPoint (Microsoft Corp, Redmond, Washington). PRISMA, Preferred Reporting Items for Systematic reviews and Meta-Analyses.

From 1123 potentially relevant articles, 17 were included in the final analysis (Figure 1). Sixteen articles were conducted in the United States and one from Singapore (Table 3). A mix of patients, their family/carers, research nurses/coordinators, physicians/oncologists, and HCLs took part in the included studies (Table 4). Five articles were from the same study population (n = 91) that included HCPs and HCLs, the majority of which were Caucasian, with 11 African American, 13 Asian, and 15 Hispanic. Most physicians and senior HCLs were men. Thirty of 33 research nurses/coordinators were women. The most common ethnic subgroups represented were African American or Black and Hispanic cancer patients. Four articles included women with breast cancer only; Trantham and colleagues’ article included only men, with the majority having prostate cancer.^19^ Remaining studies (n = 8) included a mix of oncology patients with breast, prostate, colorectal, lung, and head and neck cancers. The mean age of patients was 50+ years. Reported annual income was below $35 000 for most patients, many of whom earned >$20 000 per annum, with college-level education attainment with several patients unemployed across the articles. Most studies were undertaken in a National Cancer Institute or academic cancer center. Table 5 provides a summary of the article characteristics included in the review.

**Table 3:** Research Studies Included in Synthesis

Author (Year), Study Location and Country	Aim
Burke,^15^ (2014) Oncology safety net hospitals in North California, US	To question the importance of therapeutic misconception in the recruitment of medically underserved cancer patients to clinical research
Durant et al,^16^ (2014) Across 5 US cancer centers, US	To assess the perspectives among (1) PIs, (2) research staff, (3) referring clinicians, and (4) cancer center leaders of the barriers and facilitators to recruitment in cancer CTs
Haynes-Maslow et al,^17^ (2014) Four counties in North Carolina (urban vs rural), US	To determine the perceived barriers and facilitators to participating in cancer CTs among African American women with or who survived cancer and caregivers
Wenzel et al,^18^ (2015) Comprehensive cancer center, Baltimore, Maryland, US	To examine the processes and motivations of African American cancer patients accepting or declining participation in cancer trials
Trantham et al,^19^ (2015) Carolina Community Network, North Carolina, US	To understand the perceptions and perceived barriers to participation in cancer CT among African American men in North Carolina
Simoni et al,^20^ (2016) Across 5 US cancer centers, US	To examine motivations for minority recruitment across professional stakeholder groups (principal investigators, clinicians, research staff, and cancer center leaders)
Lee et al,^21^ (2016) National Cancer Center, Singapore	To examine the barriers to and facilitators of participation in trials among multiethnic Asian women with breast cancer
Martinez et al,^22^ (2017) Massachusetts General Hospital and Center for Community Health Education Research and Service, Boston, Massachusetts, US	To explore how Black Bostonians conceptualize barriers to cancer CTs and cancer care
Niranjan et al,^23^ (2019) Across 5 US cancer centers, US	To explore the training needs of HCPs to optimize minority recruitment and retention in cancer clinical trials
Petitte et al,^24^ (2019) West Virginia, US	To identify cultural and environmental barriers and enablers to recruitment and retention to cancer CT from patient and HCP perspectives
Niranjan et al,^25^ (2020) Across 5 US cancer centers, US	To explore how the biases of HCPs affect the recruitment and retention of racial and ethnic minority patients to cancer CTs
Rivera-Díaz et al,^26^ (2020) Cagus and San Juan, Puerto Rico, US	To identify the knowledge, motivations, and concerns influencing CT participation of Latina breast cancer patients
Wong et al,^27^ (2020) Community practices, Southern California, US	To identify barriers to participation in cancer CTs as perceived by community oncologists
Hernandez et al,^28^ (2021) Safety net hospital, Atlanta, Georgia, US	To assess barriers and facilitators to participation in cancer CT in safety net hospital. Perceptions of patient navigators to aid recruitment
Keruakous et al,^29^ (2021) National Cancer Institute cancer center, Oklahoma, US	To identify HCPs’ perspectives on barriers and facilitators to CT recruitment
Niranjan et al,^30^ (2021) Across 5 US cancer centers, US	To explore perceived institutional barriers among HCPs for minority participation in oncology CT
Legor et al,^31^ (2021) Across 10 academic medical centers (International Association of Clinical Research Nurse members), US	To develop an understanding of adult oncology CRNs’ experiences of recruiting minority patients to cancer CT

Abbreviations: CRN, clinical research nurse; CT, clinical trial; HCP, healthcare professional; PI, principal investigator.

**Table 4:** Sample Size of Included Participants Across the Included Articles

	Patients	Carers	Clinical Research Nurses/Coordinators	Physicians/Oncologists	Healthcare Leaders
Sample size (min/max)	9–39	4–45	4–33	5–50	8
No. of articles including	9	4	9 (5 of which were from the same research study)	8 (5 of which were from the same research study)	1
Total population	213	80	85	85	8

**Table 5:** Summary Characteristics of Included Articles

Author (Year), Study Location, and Country	Aim	Population (Patient, HCP, Family/Carer, Healthcare System/Provider) and Demographics	Targeted Underserved Population (ie, Ethnicity/Socioeconomic)	Methodology
Burke,^15^ (2014) Oncology safety net hospitals in North California, US	To question the importance of therapeutic misconception in the recruitment of medically underserved cancer patients to clinical research	37 oncology patients. English or Spanish. Breast and general oncology. Age range: younger, multiple comorbidities including substance abuse and mental health issues. Patient population that hospital serves is 29% Latino. 15 healthcare providers (oncologists, surgeons, fellows, residents, patient navigators, social workers and nurse practitioners)	Non-English or low English proficiency. 20 different languages. Diverse ethnicity and educational background, low SES	Ethnographic. Observations and in-depth interviews
Durant et al,^16^ (2014) Across 5 US Cancer Centers, US	To assess the perspectives among (1) PIs, (2) research staff, (3) referring clinicians, (4) cancer center leaders of the barriers and facilitators to recruitment in cancer CTs	91 participants: mean age 51. 67 Caucasian (>70%), 11 AA, 13 Asian, 15 Hispanic. 34 PIs (21 men), 8 cancer center leaders (7 men), 33 research staff (30 women), 16 referring clinicians (12 men)	Racial and ethnic minority groups. Low SES	Qualitative. Semistructured interviews
Haynes-Maslow et al,^17^ (2014) Four counties in North Carolina (urban vs rural), US	To determine the perceived barriers and facilitators to participating in cancer CTs among AA women with or who survived cancer and caregivers	82 participants. 37 cancer survivors, 43 caregivers, 2 both. Average age 57 y; 64% survivors retired or not working, 51% caregivers. 59% breast cancer and completed treatment >6 mo ago. 79% not participate in CT. 87% not informed about CT by their doctor. Cancer survivors lower educational achievement than caregivers. 48 of 70 participants earned $39 999 or less	AA women. Diverse SES (rural—less resourced and urban communities better resourced)	Qualitative. Focus groups
Wenzel et al,^18^ (2015) Comprehensive cancer center, Baltimore, Maryland, US	To examine the processes and motivations of AA cancer patients accepting or declining participation in cancer trials	32 patients (14 acceptors, 18 decliners). Median age for acceptors and decliners (58 vs 55), 12 men, 20 women. Male participants older, fewer earning <$35 000. Higher percentage of women acceptors (57%). More acceptors (n = 10) earning <$35 000. Lung, breast, prostate and colorectal cancer patients. Majority of women had breast cancer; majority of men had prostate. 7/32 were <1 y from diagnosis, 14 within 1–2 y, 5 within 2–3 y, 7 within 3–5 y	AA men and women. Diverse SES	Qualitative. Focus groups
Trantham et al,^19^ (2015) Carolina Community Network, North Carolina, US	To understand the perceptions and perceived barriers to participation in cancer CT among AA men in North Carolina	14 cancer survivors, 16 caregivers. 11 prostate cancer, 2 colon cancer, 1 oral cancer. Age 57.0 y (mean). 21 male (70%). Work full-time 12 (40%), married 20 (67%), college graduate 15 (50%), household income >40 000: 20 (67%). Have regular source of care 27 (90%)	AA men. Diverse SES	Qualitative. Focus groups
Simoni et al,^20^ (2016) Across 5 US cancer centers, US	To examine motivations for minority recruitment across professional stakeholder groups (PIs, clinicians, research staff, and cancer center leaders)	91 participants: mean age 51 y. 67 Caucasian, 11 AA, 13 Asian, 15 Hispanic. 34 PIs (21 men), 8 cancer center leaders (7 men), 33 research staff (30 women), 16 referring clinicians (12 men)	Racial and ethnic minority groups. Low SES	Qualitative. semistructured interviews
Lee et al,^21^ (2016) National Cancer Center, Singapore	To examine the barriers to and facilitators for participation in trials among multi-ethnic Asian women with breast cancer	16 participants undergoing breast cancer treatment. Age: 29–50 y = 3, 51–69 y = 13, >70 y = 0. Race: Chinese = 12, Malay = 4, Indian = 0. Education level: GCE “O” level = 3, GCE “A” level = 2, polytechnic diploma = 1, university degree = 2, unknown = 8. Stage of breast cancer: I = 4, II = 5, III = 4, IV = 3	Chinese, Malay, non-English speaking. Across various education levels	Qualitative. Focus groups
Martinez et al,^22^ (2017) Massachusetts General Hospital and Center for Community Health Education Research and Service, Boston, Massachusetts, US	To explore how Black Bostonians conceptualize barriers to cancer CTs and cancer care	All self-identified Black. 24 cancer patients (16 women and 8 men), 4 family members (all women), 5 doctors, 4 nurses (7 women and 2 men)	“Black Bostonians” including Caribbean heritage. Across local ecologies with high % of Black, (socioenvironmental, economic, histopolitical, distinct communities).	Mixed qualitative (interviews informed the development of focus group protocol)
Niranjan et al,^23^ (2019) Across 5 US cancer centers, US	To explore the training needs of HCPs to optimize minority recruitment and retention in cancer CTs	91 participants: mean age 51. 67 Caucasian, 11 AA, 13 Asian, 15 Hispanic. 34 PIs (21 men), 8 cancer center leaders (7 men), 33 research staff (30 women), 16 referring clinicians (12 men)	Racial and ethnic minority groups. Low SES	Qualitative, semistructured interviews
Petitte et al,^24^ (2019) West Virginia, US	To identify cultural and environmental barriers and enablers to recruitment and retention to cancer CT from pt. and HCP perspectives	9 patients: aged between 40–70 y; 1 colon, 9 breast; diagnosed between 1996 and 2012; 6 completed CT, 2 did not complete CT, 1 continues. 8 nurse coordinators, 10 physicians	Low SES. Racial/ethnic minorities living in rural areas	Qualitative. Focus groups
Niranjan et al,^25^ (2020) Across 5 US cancer centers, US	To explore how the biases of HCPs affect the recruitment and retention of racial and ethnic minority patients to cancer CTs	91 participants: mean age 51. 67 Caucasian, 11 AA, 13 Asian, 15 Hispanic. 34 PIs (21 men), 8 cancer center leaders (7 men), 33 research staff (30 women), 16 referring clinicians (12 men)	Racial and ethnic minority groups. Low SES	Qualitative, semistructured interviews
Rivera-Diaz et al,^26^ (2020) Cagus and San Juan, Puerto Rico, US	To identify the knowledge, motivations, and concerns influencing CT participation of Latina breast cancer patients	34 patients. Spanish speakers; 21 y and above; 62% married or cohabitating; 65% attended college or a college degree; 64% annual income below $34 999 (30%: <$15 000); 21% unemployed; 65% private health insurance—82% had difficulty with medical insurance to receive breast cancer treatment; 62% used own car for transportation; 41% had heard of CT, 32% invited to participate, 64% willing to participate in CT in future	Ethnic minority group (Latinas); majority lower SES	Qualitative. Focus groups
Wong et al,^27^ (2020) Community practices, Southern California, US	To identify barriers to participation in cancer CTs as perceived by community oncologists	20 oncologists across 6 community practices (attached to independent research and treatment center); 20% women, 65% had >10 y experience, 20% <5 y experience. 40% had <500 patients. In practice. 80% reported <5% patients enrolled in CT	Non-White race (19% of population) Hispanic (21%), non-English speaking (20%)	Qualitative. Semistructured interviews
Hernandez et al,^28^ (2021) Safety net hospital, Atlanta, Georgia, US	To assess barriers and facilitators to participation in cancer CT in safety net hospital. Perceptions of patient navigators to aid recruitment	23 patients. Mean age 57 y; 78% female; 68% diagnosed with breast ca.; remaining diagnosed with bone, lung, prostrate, colon, and nasopharynx cancer. Average time since receiving treatment was 3 y; 54.5% disabled or unemployed; 31% retired; 25% high school diploma; 20% college/technical as highest education; 56.5% annual income <$20 000, 8.7% $40 000-$60 000; 76% public insurance	AAs, lower SES	Qualitative. Focus groups
Keruakous et al,^29^ (2021) National Cancer Institute cancer center, Oklahoma, US	To identify HCPs’ perspectives on barriers and facilitators to CT recruitment	21 research nurses/coordinators (6 from gynecology, 5 breast, 3 lung and head/neck, 4 malignant hematology, 2 gastrointestinal, 1 genitourinary)	Diverse SES and underrepresented ethnic populations including AA, Black, American Indian, White alone—Hispanic/Latina (non-English—commonly Spanish)	Qualitative using Grounded theory. Survey followed by semistructured interviews
Niranjan et al,^30^ (2021) Across 5 US cancer centers, US	To explore perceived institutional barriers among HCPs for minority participation in oncology CT	91 participants: mean age 51. 67 Caucasian, 11 AA, 13 Asian, 15 Hispanic. 34 PIs (21 men), 8 cancer center leaders (7 men), 33 research staff (30 women), 16 referring clinicians (12 men)	Racial and ethnic minority groups. Low SES	Qualitative, semistructured interviews
Legor et al,^31^ (2021) Across 10 academic medical centers (International Association of Clinical Research Nurse members), US	To develop an understanding of adult oncology CRNs’ experiences of recruiting minority patients to cancer CT	19 CRN. Median age 43 y. Median years of experience in oncology = 8 y. Median years as CRN = 6 y; 18 female, 68% White, 10% Asian, 5% South Asian/Indian, 11% Black, 5% AA. Education: 68% bachelor’s, 15% MSc, 15% associate. Research setting: 90% academic medical center, 10% outpatient cancer center	Diverse ethnic minority population	Phenomenology. Qualitative, semistructured interviews

Abbreviations: AA, African American; CRN, clinical research nurse; CT, clinical trial; GCE, General Certificate of Education; HCL, healthcare leader; HCP, healthcare professional; PI, principal investigator; PS, performance status; SES, socioeconomic status; SOC, standard of care.

### Themes Derived From the Metaethnography

The third-order constructs and themes developed by the authors for this metaethnography evolved into 7 distinct categories. A narrative of the themes and constructs within the overarching 7 categories will be presented. Examples of quotations (first-order constructs) and second-order constructs from the original articles are shown in Table 6 from which third-order constructs (the authors’ interpretation) were developed. The total verbatim including metaphors and phrases included in the first- and second-order constructs was 334.

**Table 6:** Selected Quotes Illustrating Themes and Concepts

First-Order Constructs	Second-Order Constructs (Original Article Interpretation)	Third-Order Constructs (Our Interpretation) (N)	
01: “You don’t hear about clinical [trials] about cancer…I got my cousins that got cancer, but I have a cousin that she died from cancer. My…uncle, he’s currently doing a chemo, radiation, and everything, right now.”^18^^(p4)^ 02: “Use of videos will help us to understand more.”^26^^(p22)^	Lack of understanding of cancer CTs Ideal presentation of trial information	Access and source of CT information (9)	Knowledge and understanding
03: “the more research, the more possibilities there are to finding a cure….”^16^^(p1435)^ 04: “…I thank all the people who served and participated in clinical research. For example, with tamoxifen, the pill that we take in our treatments. If those women had not participated, I would not have been beneficiated….”^24^^(p6)^	Knowledge and understanding of cancer research Motivation to participate—to help others	Why research is undertaken (5)	
05: “I think it will come to a certain stage where you have no other option, when you face the wall; then you will go for the opportunity.”^26^^(p4)^ 06: “I want to decide for myself and not let luck or others decide for me.”^26^^(p6)^ 07: “Patients do not want to take the risk of being on placebo arm and would rather standard treatment.”^28^^(p4)^ Research staff 08: “… I view it sometimes as a guinea pig because you’re not sure what medicine they’ll give you. . .You never know how it’s going to affect you.”^17^^(p1098)^ 09: “It would depend on what all was involved in it. You say research but you’re not saying trial or clinical trial because if it’s just research then that doesn’t involve a clinical trial then maybe so.”^19^^(p37)^ 10: “Well one thing is … this placebo thing. I’m not a big believer in that and I think it’s sort of cruel.”^19^^(p37)^	Factors affecting decision whether to participate in CTs—factors related to the individual Perceptions and fears of cancer CTs (barrier) Concerns about CTs Factors affecting decision whether to participate in CTs—factors related to CTs Understanding of research terminology Clinical trial protocol and perception of research	Perceptions and concerns of CTs (27)	
11: “And maybe after the doctor has already explained to me, then the research assistant can give further explanations.”^26^^(p6)^ 12: “…the research team should … be orienting them about how the study is going, how are the findings; because that motivates the person to also continue her to participate….”^24^^(p6)^ 13: “…trials like that…should be offered before you go through your other stuff…But they wait until you go through all this stuff, and then offer you a trial.”^29^^(p195)^	Ideal presentation of trial information Motivation to participate Information gathering—communication; multifaceted information needs	Well informed (15)	
14: “…it should be done in a way that shows the humanized treatment given to the patients…“^24^^(p8)^ 15: “… It’s the personal- my culture being part and connecting the whole person…just acknowledging that I’m a whole person…the holistic approach, your humanness.”^17^^(p1098)^	Motivation to participate—patient and CT research team’s relationship Trust and the interpersonal aspects of care	Holistic care (3)	Interpersonal relationships
16: “I would [participate in a clinical trial] if my doctor recommended and I trust him, I would participate because I trust his medical advice.”^16^^(p1434)^	Importance of physician trust	Confidence (10)	
17: “…thanks to my higher power, which is God, that brought me more strength…It didn’t matter what direction I took, I just hold on to my faith….”^29^^(p196)^ 18: “I have a higher power, so I consider myself healed. With me, I would never participate in a clinical trial.”^16^^(p1436)^ 19: “You have to have a holistic approach doctor.. .not only do they deal with the cancer; they deal with the spirituality.”^16^^(p1434)^	Spiritual guidance Importance of faith in the decision-making process	Spirituality (9)	Patient perception and beliefs
20: “With medical research and being African Americans I know that we were used a long time ago…that’s why I think we’re so afraid of it now because they used us in the past and we don’t trust anyone.”^16^^(p1435)^ 21: “I had a lot of reservations about clinical trials… I mean look at [the] history with Tuskegee… all the different experiments that are done on Black people…That’s the only thing that holds me back….”^28^^(p5)^ 22: “…there always seems to be some kind of trial going on and, you know, in the neighborhoods of people of color….”^17^^(p1098)^	Distrust in medical system Perceptions and fears of cancer CTs Perceptions of CTs	History of abuse (7) Mistrust and Trust in research	
23: “As a Black woman, I need to know I’m not being used as a guinea pig or the financial aspects sometimes comes into play with my race in our all-Black communities.”^16^^(p1435)^ 24: “When the doctor begins getting compensation I think they may lose your best interests…“^19^^(p39)^	Distrust in medical system Relationship with physicians	Alternative motives (9)	
25: “I would go for it. If nobody takes a step, then how will medicine advance?”^26^^(p4)^ 26: “…I think I would like to try a clinical trial… it would help other women of color.”^29^^(p197)^ 27: “I see that degree of altruism more in Caucasian women than I do African American women…“^27^^(p1962)^ Referring Clinician 28: “It’s the fear, like hey if I get it I get it, I’m gonna die anyway so let me die…”	Factors affecting decision whether to participate in CTs—factors related to the individual Decision outcomes—decision-related regret Potential minority participants are not perceived to be ideal study candidates Impact of fear	Altruism (11) Fatalism (1)	
29: “I think it’s very difficult to leave your work area for a study…It would have been easier for me if the study had been at night or on a Saturday…“^24^^(p6)^ 30: “…being in a recession I was thinking about trying to get back to work…consequently I did not want to have to deal with anything that was gonna come in the way of my recuperation…“^29^^(p195)^	Motivation to participate—incentives Information gathering- economic feasibility	Other commitments (2)	Patient specific barriers
31: “…I did have some concern as to whether or not my supplemental insurance would cover what Medicare didn’t.”^29^^(p195)^ 32: “You just want to get better. You want to live.”^16^^(p1436)^	Information gathering—economic feasibility Cost and transportation would not be a barrier to participation	Cost and transport (3)	
33: “The overriding influence…didn’t come from a medical practitioner. It came from my wife who was saying, no, no, no.”^29^^(p196)^ 34: “Me and my husband and my aunt, we all decided that it wasn’t a good idea.”^29^^(p196)^	Interpersonal influences Interpersonal influences—decision partner(s)	Family influence (5)	
35: “[Patients are] recommended a clinical trial, the patients wanted it, and the HMO won’t pay for it.”^31^^(p853)^—HCP Community oncologist 36: “I think some of the protocols were very hard to do, requiring lots of patient time. Some of them required patients traveling distances. That was difficult.”^31^^(p853)^—HCP	Financial limitations Burden	Economic burden (cost and transport) (8)	HCP perception of trial burden and social determinants of health
37: “Patients often find that: a) Office visits are too much b) Laboratory tests are too frequent in between office visits c) Treatment schedules interfere with their work schedule hence affecting the quality of life and preclude financial burden.”^28^^(p4)^ CRN 38: “Trials require … perfect patients, which [are] nowhere to be found.”^31^^(p851)^	Cultural enablers and barriers—motivation Restrictive eligibility criteria	Additional trial requirements (9)	
39: “…there is an economic/educational issue which transcends the minority issue. It’s more of an economic thing… those of low education, low socioeconomic…are less trusting of being placed on a clinical trial.”^27^^(p1964)^—Referring clinician	Race was viewed as irrelevant	Socioeconomic status (5)	
**40**: “I am treating all types of cancers … there are a lot of different trials, and it is difficult to keep them all straight in my mind.”^31^^(p851)^ Oncologist	Lack of awareness of available trials	HCP knowledge and awareness of CT (11)	HCP-related factors
41: “It is much more time-efficient to…tell a patient: “[their current treatment] is not working, we’re moving to [another treatment]” … that takes 15 minutes. But if you have to go on a clinical trial … it takes double, sometimes triple the time.”^31^^(p853)^—Oncologist 42: “To get them to understand that requires a lot of ground work…and a lot of education…and the metric that is used to measure your performance is how much patients you put through the hospital…clinical trials will fall by the wayside….”^27^^(p1962)^—Referring clinician	Physician barriers—lack of time A combination of clinic-based Barriers and negative perceptions of minority study participants leads to providers with holding CT opportunities from potential minority participants	Time constraints (4)	
43: “…we all have some experience and have dealt with many different clinical situations…we have nothing formalized in terms of education or training [of minorities].”^23^^(p28)^—Principal investigator 44: “I think it would be…helpful to have sort of ongoing training…about addressing specific ways that we could improve minority recruitment into trials and retention.”^23^^(p29)^—HCP Referring clinician	Research personnel are not currently being trained to focus on recruitment and retention of minority populations Training for minority recruitment and retention provides for specific focus on factors influencing minority research participation	Appropriate training (17)	
45: “I try to recruit any patient who qualifies for the trial…I find that many of our what you call minorities…are highly educated so you just need to talk like a normal person…how would you teach a nurse or me to speak differently….”^23^^(p32)^—HCP Referring clinician	Views differ regarding the importance of research personnel training to focus on recruitment of minority populations	Refutational. Race-neutral approach (6)	
46“When a person progresses … I start thinking about switching therapy. That is one of the main…triggers to think about a trial.”^31^^(p851)^ 47: “A lack of an available standard therapy for the patient [prompts me to consider a clinical trial].”^31^^(p853)^	Lack of awareness of available trials Standard-of-care treatment no longer an option	Motivation (12)	
48: “I have had physicians that haven’t wanted non-English-speaking patients on certain [drug] trials that are quite toxic, because they worry about their ability to report adverse events.”^30^^(p3)—^CRN, White 49: “It’s just too darn hard to try to talk them into a clinical trial, so let’s just do standard therapy. The belief that they won’t follow through, they’ll be dropped out….”^27^^(p1962)^—HCP Referring clinician 50: “…I think that there’s a physician bias that’s subconscious. We know that if African Americans are offered clinical trials, their acceptance rate is about the same as the White population. …so they’re just not offered.”^27^^(p1963)^—HCL	CRN as advocate—CRNs advocate for non–English-speaking patients Potential minority participants are not perceived to be ideal study candidates Combination of clinic-based barriers and negative perceptions of minority study participants leads to providers withholding CT opportunities from potential minority participants	Bias in patient selection (12)	
51: “… when I’m getting a new patient who is a minority…a lot of times, their support system is a little more complicated…it require more thinking and more coordination.”^30^^(p4)^—CRN, Asian	CRN as care coordinator CRNs coordinate care to facilitate enrolment—CRNs coordinate care for minority patients who lack adequate social and family support systems	Role of CRN (6)	Service level factors
52: “If you have more minority faculty. That would help.”^22^^(pe670)^—HCP	Institutional efforts are needed to increase minority trial participation but those efforts are often not specific to potential minority participants	Minority HCP (2)	
53: “Most clinical trials are done in larger tertiary care centers and if you don’t get referred in then you’re not going to get access to those trials.”^17^^(p1097)^—Oncologist, Primary care	Systemic barriers in healthcare	Referrals (4)	
54: “I think access, education, informing the patients, empowering the patients…through churches…or community mechanism you have may help.”^22^^(pe670)^—Referring clinician	Cancer centers need to make consistent efforts to increase clinical research awareness in the community	Community engagement (8)	
55: “There is very little, if any direct recruitment effort to the patient. There is no aggressive recruitment effort… the information is available, but for someone who knows how to look for it.”^22^^(pe669)^—Principal investigator	There are no existing institutional programs currently being used to recruit or retain minorities to CTs	Recruitment strategies (4)	
56: “Let’s just say increase participation, period, because I don’t worry, (if) it’s minority or not.”^32^^(p7)^—HCP	Some stakeholders favor a more race-neutral approach to participant recruitment rather than an emphasis on targeted minority recruitment	Refutational—Race-neutral approach (6)	

Abbreviations: CRN, clinical research nurse; CT, clinical trial; HCL, healthcare leader; HCP, healthcare professional; N, number of quotes in this code.

#### Category 1: Knowledge and Understanding

Second-order constructs relating to patients’ and carers’ knowledge and understanding of clinical trials such as ideal presentation of trial information, motivation to participate, and perceptions and fears of cancer clinical trials were frequent concepts across the included articles. From these, third-order constructs were developed and included patient access to and source of clinical trial information, understanding of the rationale and trial-specific procedures, concerns and perceptions of clinical trials, and the need to be well informed. Understanding of clinical trials was often impacted by a lack of understanding of cancer more generally.^15^^28^ Hernandez and colleagues found some African American patients gained information through the experience of loved ones going through cancer treatment (quote 01).^28^ The mode of information delivery was important; patients, especially non-English ethnic groups such as Chinese and Malay, valued the use of picture and video information (quote 02).^21^ Opportunities to discuss clinical trials for African American patients varied depending on the physician and hospital and whether there were HCPs such as “nurse educators” and “patient navigators” available.^17^^28^ Some African American participants living in less-resourced, rural areas reported a lack of understanding of what is involved in a clinical trial and the reasoning behind trial procedures.^17^ Hernandez et al found African American patients often relied on HCPs for clinical trial information but felt this information was not always readily available.^28^ In some cases, participants could not dissociate standard of care and clinical trial treatments or had never heard of clinical trials.^28^

Healthcare professionals based in community practices frequently described their own clinical trial knowledge as a barrier (quote 39).^27^ Many found it difficult to keep a breadth of current trials and often lacked confidence in discussing them. Clinicians found institution-led initiatives to help identify suitable trials for patients; investing their time to understand the benefits of investigational drugs and discussing trials with colleagues in academic institutions were key facilitators.^22^^27^

Many African American and Hispanic patients were aware of the importance of clinical trials to advance treatments, improve patient outcomes and quality of life, and find a cure (quotes 03, 04).^17^^26^^28^ These were deemed as “patient motivators” by some primary authors. Some African American patients understood the processes involved in research, for example, allocation of standard versus trial drug and the use of animal laboratory research prior to human participatory research.^17^ Perceptions of clinical trials and associated concerns were the most reciprocal concepts within this theme.^19^^21^^22^^26^^28^^29^ Fear, mistrust, therapeutic concerns, and connotations associated with research literacy were highlighted by patients, across the ethnic groups included in the review, and often caused confusion and uncertainty.^15^^19^^21^^22^^28^

Lee and colleagues found that Asian women often perceived clinical trials as a “last resort” (quote 05) or inferior to standard treatment.^21^ Consequently, patients were often reluctant to participate if it meant a risk of less efficacious treatment. The uncertainty around randomized controlled trials and whether a patient would receive an effective treatment drug or placebo caused noticeable concern among patients and family members across several articles (quote 10).^19^^21^^24^^28^ This risk/benefit concern was also highlighted by HCPs as a significant reason for nonparticipation; they said patients, particularly those with young children, wanted a guarantee that their treatment would be effective (quote 07).^24^^29^

Connotations such as “guinea pig,” “lab rat,” “experimentation,” and “gambling” were voiced by Black and African American patients when discussing clinical trials (quotes 06, 08).^19^^21^^22^^24^^28^ Fear and uncertainty of unknown side effects, efficacy, and loss of control once on a trial meant patients dismissed or were reluctant to consider clinical trials. Healthcare professionals from large cancer centers and primary care teams were aware that the language they used could be misinterpreted by patients causing ambiguity, concern, and offense.^22^^29^ Healthcare professionals in one article highlighted that regardless of ethnicity if patients have predisposed cultural beliefs about clinical trials, they are not going to change their minds.^25^

In order to make an informed decision, African American and Latina patients expressed the need to be well informed about their cancer, treatment(s) and clinical trials (quotes 11–13), a concept echoed by HCPs.^24^^25^^26^^30^ In contrast, this need for information and time to discuss any concerns was perceived by HCPs as ethnic minority groups being less knowledgeable of clinical trials and hence portrayed in a condescending light^22^^25^: “We take a little bit more time to explain to African American [sic]I think if they have more questions because we know they are not more knowledgeable, so I think it takes time”^25^^(pp1960–1961)^—principal investigator.

The volume and timing of information about clinical trials were highlighted among Asian and African American patients.^18^^21^ Patients benefited from hearing about clinical trials at diagnosis to allow them to process it before “the emotion of it all takes over.”^21^ Patients felt open and honest dialect with HCPs was crucial.^18^^26^ Healthcare professionals, including clinical research nurses (CRNs) from cancer centers, agreed communication was a key facilitator to trial participation.^16^^31^ Principal investigators from US cancer centers successful in recruiting ethnically diverse groups argue that framing the notion of clinical trials as a treatment option and not as a last resort increases the likelihood of patients participating in clinical trials.^30^ However, HCLs from the same study argue that HCPs often underplay the benefits of clinical trials.

#### Category 2: Interpersonal Relationships

This category highlights the second-order constructs that include the patient and clinical trial team’s relationship, the trust between the patient and HCP, and interpersonal aspects of care. The levels of trust patients instill in their HCP and how those influence patients’ decision-making are portrayed. Several patients identified the importance of a “humanized approach” when discussing clinical trials (quotes 14, 15).^26^ Clinical research nurses often provided interpersonal care and went that “extra mile” to make sure patients were supported throughout their cancer treatment.^24^ However, Martinez and colleagues found examples of poor interpersonal care received by Black patients^22^: “He just was very non-personal, very reserved, critical after the surgery… and would only come by once and say something negative and critical like: how come you’re always sitting down?”^22^^(p1098)^—Black Bostonian.

Patients’ confidence and trust in the physician’s medical knowledge was crucial among African American and Asian patients (quote 16).^17^^19^^21^ Some patients preferred senior physicians to give information rather than any other HCPs.^24^ Others felt their physician knew their cancer and health condition better than anyone and therefore were self-assured with their physicians’ treatment advice.^17^^21^^24^ This trust and confidence patients instill in HCPs to make healthcare decisions were identified by HCPs themselves: “The physician is the person our patient most relies on to make informed decisions on their healthcare”^29^^(p4)^—CRN.

Some HCPs reported challenges in building a trusting rapport with some ethnic minority groups. In Niranjan and colleagues’ study, HCPs who were majority White/non-Hispanic argued that certain minority patients had “distinct temperaments,” which made the recruitment process “arduous.”^25^ According to Martinez et al, HCPs felt patients from Black communities often had a better rapport and trusting relationship with their primary care physicians rather than with oncologists at large cancer centers.^22^

#### Category 3: Patient Perception And Beliefs

This category encapsulates second-order constructs that represent participants’ perceptions and beliefs that impact decision-making. These include spiritual guidance, the importance of faith and altruism, the impact of fear, and the distrust in the medical system. From this, third-order constructs were developed including spirituality, fatalism, history of abuse, mistrust, and trust in research and altruism. Spirituality was a reciprocal finding across 5 articles and within the Black/African American participants.^17^^18^^19^^24^^29^ Patients discussed a spiritual connection with a higher power based on prayer and God, which would help them cope with their cancer diagnosis or give them the strength to make treatment decisions (quotes 17, 18). Caregivers also spoke of the importance of spirituality and praying to “instill everything in that doctor.”^17^^(p1436)^ The importance of having a physician who appreciates a patient’s spirituality was highlighted (quote 19).^17^ Only one article found Black Bostonian patients to express a fatalistic approach to cancer diagnosis, which may preclude thinking about treatment including clinical trials (quote 28).^22^

Patients commonly referred to examples of historical abuse within medical research and clinical trials causing fear and mistrust among Black/African American participants (quotes 20, 21).^17^^18^^26^^29^ Human experimentations such as The Syphilis Study at Tuskegee, the commercial appropriation of Henrietta Lacks’ tumor cells, and what occurred in the Nazi concentration camps were often mentioned. Hernandez et al found that older male African American patients reported greater mistrust and fear of participating in clinical trials.^28^ However, several studies resonate the historical abuses reported across generations, representing the intersectionality of participants, irrespective of age and sex. Some HCPs working in primary care correlated patients’ mistrust and “skepticism” in medical research around cultural attitudes and beliefs and were sometimes referred to as a “patient barrier” by HCPs, without appreciating the roots of the cause.^22^^27^

Distrust and weariness that only certain ethnic groups are targeted to take part in cancer clinical research were felt by a number of Black/African American participants (quote 22).^17^^22^ To help reassure and make patients feel more comfortable participating in clinical trials, patients did not want to be identified as a race or ethnic group.^17^ Black, African American, and Latina patients expressed concern that clinicians have alternative motives for recruiting ethnic minority groups, such as financial gains, rather than putting the interests of patients first (quotes 23, 24).^17^^18^^19^^26^ Refutational to this were African American women who associated financial gains with a guarantee that physicians would be more diligent and therefore were more likely to participate.^17^ In addition, compensation was acceptable if clinicians were forthcoming about it. Refutational to common concepts around patients’ fear and distrust in medical research, in an article by Lee and colleagues, Chinese and Malay patients expressed their trust in the drug development process and government legislation.^21^

Across all ethnic groups included in the studies, altruism was a reciprocal theme associated with a patient’s incentive to participate in a clinical trial.^17^^18^^19^^26^ Patients recognized the value of trial participation for “the greater good” and to help others (quote 25). Personal experience of having family members with cancer or undergoing investigations/treatment for cancer and the difficulties associated with this were frequently expressed.^26^ African American patients described a “health disparities dilemma” where they compared and contrasted feelings of altruism with research distrust. Patients acknowledged a personal responsibility to participate to help ameliorate ethnic minority health and treatment disparities (quote 26).^18^ Contrary to the importance of altruism in patients’ decision-making, this was not perceived by HCPs. Instead, Niranjan and colleagues found HCPs to have a stereotypical view that certain ethnic groups were more altruistic than others (quote 27).^25^ Patients’ personal incentives of accessing new drugs to improve their health and a perceived expectation of preferential treatment were voiced by HCPs across 5 cancer centers.^25^

#### Category 4: Patient-Specific Barriers

This category incorporates second-order constructs from 3 articles including economic feasibility and interpersonal influences/decision partners.^17^^18^^26^ These were synthesized to represent third-order constructs that focus on interpersonal relationships with family and socioeconomic factors affecting mainly African American patients’ decision to participate in clinical trials. Patients’ family circumstances and the role of the family, referred to as decision-partners by one author,^18^ contribute to patients’ decision-making (quote 33). Concordance between ethnically diverse patients and “decision-partners” was important, particularly when patients were undecided (quote 34).^18^ Even when patients reported a positive interpersonal relationship with their doctor, family and friends often had a stronger and more direct influence on patients’ overall decisions.

The decision to participate was also influenced by the frequency of additional trial visits, additional tests/procedures, employment status, work and family commitments, and transportation combined with associated financial implications (quotes 29, 30).^18^^26^ Patients’ concerns around the lack of medical insurance plans covering the additional investigations required in clinical trials caused patients to decline or be excluded by HCPs (quote 31).^18^ Although 2 studies identified the socioeconomic burden as a tangible barrier to clinical trial participation, this was not a prominent reciprocal theme expressed by patients across the included articles. Many African American women in Haynes-Maslow and colleagues’ study said they would do “whatever it takes”^17^ to get the best treatment to survive.

#### Category 5: Healthcare Professionals’ Perceptions of Trial Burden and Social Determinants of Health

This category aims to identify what HCPs perceive to be the main patient-related barriers to and facilitators of participating in cancer clinical trials. Three main themes were identified using second-order constructs across the included articles, which include economic burden, additional trial requirements, and socioeconomic status. Many HCPs report the socioeconomic burden associated with ethnic minority participation to clinical trials as a patient-reported barrier (quotes 35, 36).^16^^24^^25^^27^^29^ Healthcare professionals acknowledge the importance of ensuring patients are fully aware of what is required and the potential impact on them financially.^16^^24^^27^ Healthcare professionals reported enablers such as providing financial support directly from the institution or from funding groups or using outreach models to address financial concerns.^24^

Several HCPs from both cancer/academic centers and community hospitals felt patients from ethnic minorities had concerns about rigid trial protocols and additional invasive procedures that may dissuade them from participating (quotes 37, 38).^24^^27^^29^ A reciprocal theme across 2 articles involving HCP participants was the belief that deep-rooted barriers associated with education and socioeconomic status contributed more than ethnicity to patient participation (quote 39).^23^^25^ One HCP maintained that upper-middle-class patients, irrespective of race, would not differ from other patients belonging to the same economic status; therefore, socioeconomic status overrides race in shaping the perceptions of trial participation.^25^ Similarly, Niranjan and colleagues reported minority patients are unable to navigate through the complex healthcare systems associated with academic medical centers due to socioeconomic factors.^30^

#### Category 6: Healthcare Professional–Related Factors

This category aims to describe HCP-reported barriers that often influence clinical trial enrollment for ethnic minorities. Second-order constructs around lack of awareness of available trials (quote 40), clinic-based barriers, training, and negative perceptions of minority study participants were common across the articles. From these, third-order constructs were synthesized, which include HCP knowledge and awareness of clinical trials, time constraints, appropriate training, race-neutral approach, and bias in patient selection. Community oncologists’ time constraints were a prominent theme.^22^^24^ The time required to identify a trial, assess eligibility, and discuss the trial with ethnically diverse patients was seen as onerous, and therefore, standard of care was viewed as more time-efficient and practical (quote 41).^27^ Within larger cancer centers, HCPs believed that limited physician time and external pressures made it burdensome to discuss the complex nuances of clinical research (quote 42).^25^ Despite time restraints, HCPs noted that being prompted by the patients or fellow research staff was a facilitator.^27^

Healthcare professionals from both community and cancer/academic centers involved in clinical trials described a lack of cultural awareness training (quote 43).^23^^27^^30^ Training rarely focused on recruiting ethnic minority populations, and if available, it was described as minimal and/or inadequate.^23^ Cultural competency training is required to understand ethnic minority decision-making and how health-seeking behaviors are influenced by unique factors. Learning through experience or via trial and error was believed not to be beneficial or recommended.^23^ There was an assumption that HCPs with sufficient years of general oncology experience and training would be able to use their skills to recruit from all ethnicities.^23^ Healthcare professionals in Niranjan’s article acknowledged that drawing from their own life experiences as well as from colleagues of different ethnicities helped their understanding and recruitment of patients from ethnic communities.^23^

Information and training on historical and cultural perceptions and beliefs and practices of different ethnic communities alongside the structure and function of family units was deemed valuable for HCPs (quote 44).^23^ Healthcare professionals noted that understanding such nuances and using culturally sensitive language would be beneficial, especially during sensitive discussions such as a man’s sexual function following prostate cancer treatment.^23^

Concerns around interpreters’ interactions and clinical trial information being “lost in translation” were voiced by HCPs across 5 cancer centers in the United States.^23^ Interpreters trained in cultural awareness were recommended. A further concern was the potential for an “imbalance” in training, with an extreme focus on ethnic diversity and inclusion in clinical trials at the expense of excluding the fundamentals of human subjects’ recruitment and ethical principles, consequently creating an unskilled research workforce.^23^ Refutational to the need for cultural competency training, Niranjan and colleagues highlight the belief that focused training for the recruitment of ethnic minorities was not necessary.^23^ Some HCPs believe trial underrepresentation does not exist, and therefore, training should consist of a blanket approach for all patients, regardless of race or ethnicity. The use of clear communication in layman’s terms obviated the need for specific training (quote 45). This race-neutral approach was deemed effective as it promoted no differentiation between racial and ethnic groups.^23^

Healthcare professionals discussed various incentives for recruiting ethnic minorities into clinical trials. For HCPs working at National Cancer Institute cancer centers, these include maintaining or achieving academic status and scientific leadership through adherence to federal guidelines, which mandate the inclusion of ethnic minorities to cancer clinical trials; receiving funding; and personal experiences of cancer and racial health disparities in treatment.^20^ Patients’ stage of disease (quote 46), the availability of standard-of-care treatments (quote 47), and the perception that patients taking part in clinical research have better outcomes were perceived as enablers by community HCPs.^27^ However, structural factors facilitating reimbursement for routine clinical care were seen to have a negative impact on HCPs’ priorities, motivation, and allocation of time for ethnic minority recruitment in trials: “I get more relative value if I… ask about 10 organ systems, I get nothing for asking about clinical trials or exploring preferences…If I spend my time talking about clinical trials… then I’ve reduced the complexity of the visit”^30^^(p669)^—principal investigator.

Synthesizing first- and second-order constructs across the included articles revealed examples of bias and stereotyping among HCPs in their decision-making and enrollment of ethnic participants in clinical trials.^15^^16^^25^^31^ Burke revealed HCPs’ dismissive, patronizing behaviors if patients of ethnicity expressed concerns around trial therapies, causing therapeutic misconceptions.^15^ Healthcare professionals voiced non-English communication as a barrier, and perceived patients will have a poor understanding of complex protocol requirements.^16^^25^ There was a preconceived notion that language barriers would also cause trial drug compliance concerns and safety risks due to poor reporting and not knowing what to do if adverse events occurred (quote 48).^25^^31^ Healthcare professionals reported that certain ethnic groups are more compliant than others.^25^ Such nonadherence prejudice was perceived as burdensome to research teams, as reporting protocol deviations to study sponsors would cause additional workload (quote 49).^25^ Unconscious bias shown by HCPs around language barriers was a reciprocal theme whereby use of translators, additional consultation time, and the expense of recruitment materials in multiple languages were deemed as troublesome.^16^^25^ For patients with basic healthcare insurance in the United States, there was concern that additional trial costs would be incurred and that therefore it would be easier to exclude patients from ethnic minority groups with lower socioeconomic status.^25^ Verbatim from Niranjan and colleagues’ article illustrates research staff, and HCLs acknowledge that unconscious/conscious bias exists (quote 50).^25^ Hence, shared decision-making is not achieved or even initiated as standard of care is deemed less taxing and less expensive. One HCP acknowledged their discomfort around establishing an interpersonal relationship in order to establish trust with ethnic minority patients.^25^

#### Category 7: Service Level Factors

This category represents HCPs’ and HCLs’ perspectives on service-level factors that can impact ethnic minority recruitment into cancer clinical trials including recruitment strategies. Third-order constructs were synthesized from second-order constructs across 8 articles.^16^^22^^24^^25^^27^^29^^30^^31^ These include the roles of the CRN, minority HCPs, referrals, community engagement, and the refutational concept of a race-neutral approach. Oncologists working in the community described increasing clinical workload with insufficient infrastructure support including the number of clinically trained staff, which was exemplified by the impact of the COVID-19 pandemic.^27^ The impact of COVID was a reciprocal theme in Keruakous and colleagues’ article.^29^ Clinical research nurses across 10 cancer institutions in the United States argued that their role was potentially under threat as many research centers are moving toward recruiting unlicensed research coordinators to reduce costs.^31^ They argue that lack of clinical knowledge among research coordinators will impact patient care, and trial recruitment and retention may be compromised. Clinical research nurses play a significant role in recruiting ethnic minority patients into clinical trials. They coordinate interpreters, provide support with financial issues, and assist patients who have inadequate social support (quote 51). Clinical research nurses frequently observed inadequate family/social support systems among ethnic minority patients.^31^

Increasing the ethnic diversity research workforce was viewed by HCPs in cancer/academic centers as an enabler to increase clinical trial participants across ethnic communities (quote 52).^30^ Having a comprehensive trial portfolio was deemed a main facilitator for ethnic minority recruitment.^27^ However, in the community, HCPs reported a reduced number of suitable trials compared with larger academic sites.^27^ Strict eligibility criteria were also a significant recruitment barrier.^29^ Healthcare professionals from larger academic sites suggested simplifying and streamlining referrals between community hospitals and cancer centers with additional support for minority patients to help navigate between care providers (quote 53).^22^^30^ To improve awareness, referrals, and access to clinical trials, HCPs highlighted the importance of networking, establishing links, and building rapport with colleagues working within communities of specific ethnic groups.^25^ A reciprocal concept was the need for increased engagement and outreach models of clinical trial delivery across ethnic minority communities (quote 54).^16^^30^^31^

The lack of existing or planned institutional strategies for ethnically diverse recruitment was raised by research staff across 4 cancer centers.^25^^30^ Despite the availability of information on cancer clinical trial opportunities, there was a lack of tailored initiatives to address the recruitment of ethnic minorities (quote 55). It was generally felt that without strategic funding, resources, and incentives, inequalities will continue. Healthcare professionals argue that the “clinical research enterprise” is not designed for clinicians to focus on minority recruitment, but instead on the set targets of clinical productivity: “…you can have all of the cultural competency in the world if you have a small, completely underfunded, hard-to-manage, clinical research enterprise with physicians who need to do something else to make money. You’re not gonna put anybody in a trial even with the best of intent”^30^^(p669)^—HCL.

Refutational to the need for minority recruitment strategies, few HCPs across 5 US cancer centers had a “race-neutral” perspective where race was not seen as a contributing factor.^20^^23^^25^ Hence, no specific considerations or special efforts toward minority recruitment are required. All human subjects should be equally prioritized rather than focusing on or targeting specific groups; therefore, a “personalized” blanket approach is required (quote 56).

**Figure 2:**
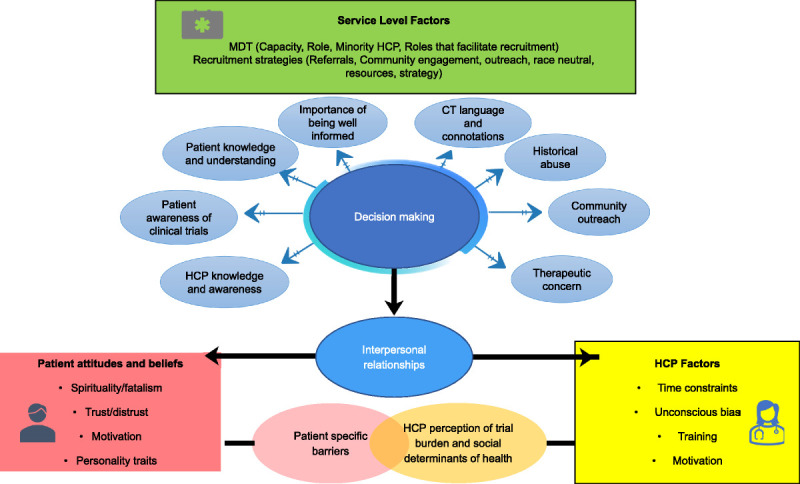
Conceptual framework of the barriers and enablers. Figure designed using Microsoft Office PowerPoint (Microsoft Corp).

A conceptual model to illustrate the multifaceted barriers and enablers identified was created (Figure 2). The researcher considered their own experiences when developing the conceptual framework and from this developed 4 lines of argument. An updated search was carried out between February 1, 2022, and January 31, 2023, which found reoccurring themes to support the findings of this metaethnography.^33^^34^^35^

## Discussion

To inform equity, diversity, and inclusion strategies, it is paramount to gain knowledge and understanding of the existing literature. To our knowledge, this is the first systematic review from a metaethnography theoretical standpoint including ethnic minority patients, their carers, HCPs, and HCLs. The review findings reveal important contextual detail to inform our understanding of the barriers and enablers to participation in oncology clinical trials across the included ethnic minority communities. This is a valuable contribution to the literature as it represents the perceptions and views of those who receive healthcare and those who provide it. It highlights the incongruities between patient-reported barriers and those perceived by HCPs, adding complexity to what is already a multifaceted dilemma.

Central to this systematic review has been patients’ knowledge and understanding of clinical trials. It is well reported in the literature that increased awareness and knowledge of the rationale, benefits, and processes involved in clinical trials can encourage participation by alleviating uncertainties and concerns.^8^^36^ Communication efforts by HCPs and the research literacy used are often ineffective because HCPs neglect cultural, social, psychological, environmental, and historical factors that influence patients’ perceptions of health and clinical research.^33^ Research literacy can instill fear, distrust, skepticism, and misperceptions, which then impact patient decision-making. Hecht and Krieger argue that the need to communicate from a culturally grounded perspective is paramount.^37^ The importance of an interpersonal relationship between the HCP and the patient and family/carer was unnoticed by the HCP. Few patient-focused interventions designed to improve doctor-patient interpersonal discussion of clinical trials appear in the literature. Social psychology research suggests that patients and physicians in racially discordant clinical interactions should create a sense of common purpose, support, and understanding as ways to reduce bias and increase a therapeutic and trusting relationship.^38^ Increasing inclusivity from differing ethnic backgrounds in the medical research workforce, could play a significant part in facilitating this.^35^ Having a diversified workforce who are bilingual and bicultural has been reported to help increase ethnic minority recruitment.^39^

This metaethnography demonstrates the multiple components that intersect across both patients and HCPs, which impact ethnic minority patients accessing and participating in clinical trials. There is an unconscious and conscious bias that exists within healthcare and by HCPs that has a significant impact on patients’ awareness, opportunities, and acceptance of cancer clinical trials.^33^^40^ This is supported by earlier research showing physicians displaying negative attitudes toward African Americans, leading them to believe they will be poor candidates for clinical trials or less likely to participate.^41^ Conversely, studies have shown that African Americans have an interest in participating in research, more than any other racial and ethnic group.^42^^43^ Penner and colleagues found that oncologists with higher rates of implicit bias used less patient-centered communication.^44^

Due to the negative effects of unconscious bias, patient mistrust in medical research can often be perceived by HCPs as reluctance to participate.^45^ A recommendation would be for all HCPs to undertake cultural safety training, which requires HCPs to examine themselves and the impact of their own culture on clinical interactions instead of HCPs focusing on learning cultural customs of different ethnic groups.^46^ According to Laverty and colleagues, cultural safety is about acknowledging the barriers to clinical effectiveness arising from inherent power imbalance between provider and patient.^47^ Cultural safety seeks to achieve better care through being aware of the differences, decolonizing, considering power relationships, implementing reflective practice, and allowing the patient to determine whether a clinical encounter is safe.^46^ The metaethnography illustrates a discordance between patient-reported barriers and what HCPs believe are the main patient barriers. The importance of social support and family involvement, patients’ spirituality and altruism in the decision-making process were not identified by HCPs as contributory factors in cancer clinical trial participation. Somayaji and Cloyes found that many clinical trial participants reported a “spiritual connection with a higher power” that led them to participate.^48^ Four religious coping approaches have been theorized with various levels of individual control: deferring (passive role leaving the entire decision to God), pleading (requesting God’s intervention), self-directing (patient decision but God will support them), and collaborating (God as a supportive partner).^49^ Few examples of these religious coping approaches can be seen within this metaethnography. Future research into the relationship between the individual and their spirituality may help to capture the effects of religious beliefs and distinguish subgroups of people among whom religious beliefs may or may not play a significant role in clinical trial participation decision-making.

A coproduced robust Equality, diversity and Inclusion strategy is needed to address inequalities in clinical trial participation. Expanding clinical research beyond the larger academic cancer centers allows access to a wider and more diverse population, accelerates accrual, and can increase the generalizability of trial findings. Previous research has shown that community health centers in the United States improve access to high-quality healthcare and are effective in reducing disparities.^50^ The race-neutral approach by some HCPs/HCLs disregarding the need for tailored recruitment strategies is an important finding. Such ideologies deter the focus away from the deep-rooted problems that exist and have detrimental effects on both patients as well as policy makers. The myriad factors that influence diversity among individuals and groups such as language, nationality, acculturation, education, literacy, socioeconomic status, religion, spiritual and health beliefs, and practices will be ignored.

### Strengths and Limitations

This metaethnography is the first analytical evaluation of qualitative data around the barriers to and facilitators of cancer clinical trial participation from both the patient and HCP/HCL perspectives. It highlights the multifaceted elements that are cross-cutting between patients’ and HCPs’ experiences as well as illustrating the contradictions that exist. All but one article was published in the United States, with English-speaking minority groups, predominantly women of African American or Black ethnicity. Signifying that even within the United States the included articles do not mirror the diversity of its population, indigenous populations are particularly underrepresented in clinical trials. American Indian/Alaska Natives have the lowest representation of racial/ethnic groups in the United States.^32^ Therefore, the barriers and facilitators identified may not be generalizable across other ethnic minority groups or healthcare systems. Men and women have different perspectives on healthcare, as well as generational differences that have not been well represented in this review. The research examined does not acknowledge the intersectionality and heterogeneity among ethnic groups that exist or how faith is related to an individual’s health beliefs. The included research was mainly carried out in large academic cancer centers and hence may not represent the institutional and system-related barriers that exist in community-based hospitals. Healthcare professionals and HCLs were majority White, with physicians mainly male. Therefore, institutional biases and culturally naive perspectives may hinder reported barriers. The researcher acknowledges that her extensive career in breast oncology research may influence the interpretive process by subjectively bringing in her own perceived barriers and enablers. However, other members of the research team were able to bring an objective approach from a purely academic perspective.

## Recommendations

An EDI strategy should address (1) health literacy and communication including allowing sufficient time, appropriate use of culturally sensitive language, adequate provision of interpreters, information in various languages, and sufficient training for HCPs; (2) continual community engagement to understand local barriers, build trust, and inform communities of the role and benefits of clinical trials, as well as outreach models of care to provide equity of access, deliver clinical trials closer to home, and address socioeconomic burden; (3) utilizing dedicated roles within MDTs, including CRNs and patient navigators to provide holistic support, ensure trial information and opportunities are communicated to patients effectively, and increase the knowledge and confidence of clinical teams. Oncology nurses are perfectly placed to identify existing barriers for ethnic minority patients as well as implement and evaluate relevant and meaningful EDI strategies. The findings reaffirm the importance and value of interpersonal skills and delivering care in a “humanized” and holistic approach, skills that underpin cancer nursing. Strong interpersonal skills promote collaboration, trust with patients, and self-awareness, all of which have been highlighted in this review as being pertinent to address the barriers that have been discussed.

It is paramount that all levels of oncology nurse training programs promote the importance of cancer clinical trials, their role in addressing the inequalities that exist, and applying cultural safety as a core principle to their practice. This will enable nurses to acknowledge and address their own biases, attitudes, assumptions, stereotypes, and prejudices that may influence the quality of care they provide. In addition to oncology nurses and wider HCP education and training in cultural safety, it is essential that healthcare organizations (and society at large) can have culturally safe environments to achieve health equity.^46^

A prominent theme throughout this meta-analysis is the need to address research literacy, cultural safety, and unconscious biases within healthcare and among HCPs/HCLs. This systematic review highlights the detrimental effects that such themes can lead to ethnic minorities being less likely to receive information or, even worse, labeled as unwilling to participate. If steps are not taken to address these, then overcoming barriers such as distrust, fears, and misperceptions associated with clinical trials will prove challenging.
